# Ethnic-Specific Differences in Vitamin D Status Is Associated with Adiposity

**DOI:** 10.1371/journal.pone.0043159

**Published:** 2012-08-29

**Authors:** Dian C. Sulistyoningrum, Timothy J. Green, Scott A. Lear, Angela M. Devlin

**Affiliations:** 1 Department of Pathology and Laboratory Medicine, University of British Columbia, Child and Family Research Institute, Vancouver, Canada; 2 Department of Food Nutrition and Health, University of British Columbia, Child and Family Research Institute, Vancouver, Canada; 3 Faculty of Health Sciences, Simon Fraser University and Division of Cardiology, Providence Health Care, Vancouver, Canada; 4 Department of Pediatrics, University of British Columbia, Child and Family Research Institute, Vancouver, Canada; Wageningen University, The Netherlands

## Abstract

**Background:**

Low circulating 25 hydroxyvitamin D [25(OH)D] concentrations are common in obesity (BMI ≥30 kg/m^2^) and a negative relationship with body fat distribution has recently been reported. Ethnic-specific differences in body fat distribution have been described with South Asians are reported to have greater visceral adipose tissue (VAT), which could influence circulating 25(OH)D concentrations. The objective of this study is to investigate the relationship between plasma 25(OH)D, adiposity, and body fat distribution in Europeans and South Asians.

**Methods/Principal Findings:**

187 Europeans and 192 South Asians were assessed for demographics, anthropometrics, and plasma 25(OH)D concentrations. Subcutaneous adipose tissue (SAT) and VAT were quantified by CT scan, and percent body fat by DEXA. Data were assessed by general linear models. South Asians had lower (*P*<0.001) plasma 25(OH)D concentrations and higher VAT (*P* = 0.04) than Europeans. Plasma 25(OH)D concentrations were negatively (*P*<0.05) associated with BMI, waist circumference, percent body fat, total adipose tissue, VAT, and SAT in unadjusted models and negatively (*P*<0.05) associated with VAT, SAT, and percent body fat after adjusting for BMI, ethnicity, age, and season of blood collection in males and females. When percent body fat, VAT, and SAT were included in the same model, only VAT remained negatively (*P*<0.05) associated with plasma 25(OH)D concentrations. Ethnicity remained significant in all models (*P*<0.001).

**Conclusion:**

Compared to other adipose tissue compartments, VAT may have a distinct role in determining plasma 25(OH)D concentrations, which may account for the lower levels in South Asians.

## Introduction

Low vitamin D status is associated with a number of adverse health outcomes including osteoporosis, certain cancers, autoimmune conditions, and more recently cardiovascular disease (CVD) [1]. Vitamin D status is best assessed by quantifying circulating 25 hydroxyvitamin D [25(OH)D] concentrations. Low vitamin D status has been defined by the Institute of Medicine as a circulating 25(OH)D concentration <50 nmol/L [2]; although a concentration of 70–100 nmol/L has been suggested for optimal health [3]. Vitamin D can be obtained from a small number of foods and through skin synthesis by the action of UV light. Factors that affect the amount of UV light reaching the skin include age, sex, season, latitude, and skin colour, and thereby influence circulating 25(OH)D concentrations [4]–[7].

Obesity, defined as a BMI ≥30 kg/m^2^, has been reported to be inversely associated with circulating 25(OH)D concentrations [4], [8], [9]. Initially it was suggested that this association was due to limited outdoor physical activity and hence, less sun exposure in these individuals [10]. However, adipose tissue may sequester vitamin D resulting in decreased bioavailability [11], [12]. In support of this, a negative relationship between subcutaneous adipose tissue (SAT) and visceral adipose tissue (VAT) deposition and serum 25(OH)D concentrations has been reported in an American Caucasian population [10] and in Hispanic American and African American populations [13]. Given that VAT deposition is an independent risk factor for CVD [14] and low circulating 25(OH)D concentrations are also associated with CVD [10], low circulating 25(OH)D contributions may be another factor that contributes to the pro-atherogenic risk of excess VAT.

A small number of studies have identified that South Asians are more prone to low circulating 25(OH)D concentrations than individuals of European descent, presumably because of their darker skin colour [15]–[19]. These studies however, have been small and have not investigated other possible determinants. Other factors, such as excess VAT deposition, may be contributing to circulating 25(OH)D concentrations in South Asian populations.

The Multicultural-Community Health Assessment Trial (M-CHAT) found that South Asian subjects have higher amounts of body fat and VAT compared to European subjects of the same body mass index (BMI) and body fat mass, respectively [20]. As shown for an American Caucasian population [10], the higher amounts of total body fat and VAT in South Asian subjects may contribute to the lower circulating 25(OH)D concentrations observed in South Asian populations [15]–[19]. As such, the goal of this study is to investigate if plasma 25(OH)D concentrations are associated with body fat composition in European and South Asian subjects from the M-CHAT cohort.

## Results

Ethnic-specific differences in demographics, anthropometrics, and body fat distribution are shown in Table 1. South Asian subjects were younger (*p*<0.001), and had higher amounts of total abdominal adipose tissue (*p* = 0.038), VAT (*p* = 0.04), and percent body fat (*p* = 0.001) than Europeans. Plasma 25(OH)D levels were lower (*p*<0.001) in South Asian than European subjects (42.26±16.6 nmol/L vs 67.42±26.0 nmol/L, respectively). There were also a lower percentage of samples collected during the summer months (July to October) from European subjects than South Asian subjects (29.7% vs 41.0%, respectively, *p* = 0.008).

**Table 1:** Characteristics of subjects by ethnicity.

	European (n = 182)	South Asian (n = 188)	Significance
Women (%)	91 (49)	93 (47)	
Season of blood collection (%)			*p* = 0.008
Nov–Feb	32 (17.6)	42 (22.3)	
Mar–Jun	96 (52·7)	69 (36·7)	
Jul–Oct	54 (29·7)	77(41·0)	
Age (years)	50.70±9.14	44.97±8.36	*p*<0.001
BMI (kg/m^2^)	27.78±5.08	27.86±4.96	*p* = 0.904
Waist circumference (cm)	89.65±12.7	88.76±12.3	*p* = 0.489
Waist/hip ratio	0.875±0.09	0.883±0.10	*p* = 0.410
Total abdominal adipose tissue (cm^2^)	411.8±177	448.9±165	*p* = 0.038
Subcutaneous adipose tissue (cm^2^)	266.7 (197, 386)	309.4 (224, 391)	*p* = 0.030
Visceral adipose tissue (cm^2^)	102.1 (79, 145)	118.7 (88, 162)	*p* = 0.015
Total body fat (%)	32.49±9.95	35.88±9.26	*p* = 0.001
Plasma 25(OH)D (nmol/L)	67.42±26.0	42.26±16.6	*p*<0.001

Values presented are means ± SD. Subcutaneous and visceral adipose tissue were not normally distributed and were transformed using the natural log for statistical analyses; values presented are the median (25^th^ percentile, 75^th^ percentile). Significant differences between ethnic groups were assessed by ANOVA. Ethnic-specific differences in season of blood collection were assessed by Pearson's chi-squared test.

We analyzed the relationship between plasma 25(OH)D concentrations and anthropometric measures and body fat distribution. Plasma 25(OH)D concentrations were negatively associated with BMI (*p*<0.001), waist circumference (*p* = 0.015), total abdominal adipose tissue (*p*<0.001), VAT (*p*<0.001), SAT (*p*<0.001), and percent total body fat (*p*<0.001) (Table 2).

**Table 2:** Relationship of plasma 25(OH)D with anthropometric measures and body fat distribution.

	R-value	Significance
BMI	−0.195	*p*<0.001
Waist circumference	−0.131	*p* = 0.015
Waist/hip ratio	−0.056	*p* = 0.302
Total abdominal adipose tissue	−0.312	*p*<0.001
Ln VAT	−0.285	*p*<0.001
Ln SAT	−0.289	*p*<0.001
Total body fat	−0.307	*p*<0.001

Data were analyzed using unadjusted separate linear regression models.

SAT, subcutaneous adipose tissue; VAT, visceral adipose tissue.

In women, plasma 25(OH)D concentrations were negatively associated with total abdominal adipose tissue (*p* = 0.002), VAT (*p*<0.001), SAT (*p* = 0.042), and percent total body fat (*p* = 0.008) in separate models after adjusting for BMI, ethnicity, age and, season of blood collection (Table 3). In all of these models, ethnicity remained a significant contributor (*p*<0.001) such that South Asians had lower plasma 25(OH)D concentrations than the Europeans. In men, plasma 25(OH)D concentrations were negatively associated with VAT (*p* = 0.001) and percent body fat (*p* = 0.025) in separate models after adjusting for BMI, ethnicity, age, and season of blood collection (Table 3). As in women, ethnicity remained significant (*p*<0.001) in all models, with plasma 25(OH)D concentrations lower in South Asian than European male subjects. The strongest associations in both women and men were observed between VAT and plasma 25(OH)D concentrations [standardized β-coefficients −0.354 (*p*<0.001) and −0.256 (*p* = 0.001), respectively].

**Table 3:** Relationship of plasma 25(OH)D with anthropometric measures and body fat distribution in men and women.

	Females		Men	
	β-coefficient (standardized)	Significance	β-coefficient (standardized)	Significance
Waist circumference	−0.173	*p* = 0.193	−0.099	*p* = 0.492
Total abdominal adipose tissue	−0.492	*p* = 0.002	−0.199	*p* = 0.067
Ln VAT	−0.354	*p*<0.001	−0.256	*p* = 0.001
Ln SAT	−0.258	*p* = 0.042	−0.122	*p* = 0.195
% Total body fat	−0.288	*p* = 0.008	−0.197	*p* = 0.025

Data were analyzed by separate linear regression models adjusted for ethnicity, age, BMI, and season of blood collection. Females and males were analyzed separately.

SAT, subcutaneous adipose tissue.

VAT, visceral adipose tissue.

**Figure 1:**
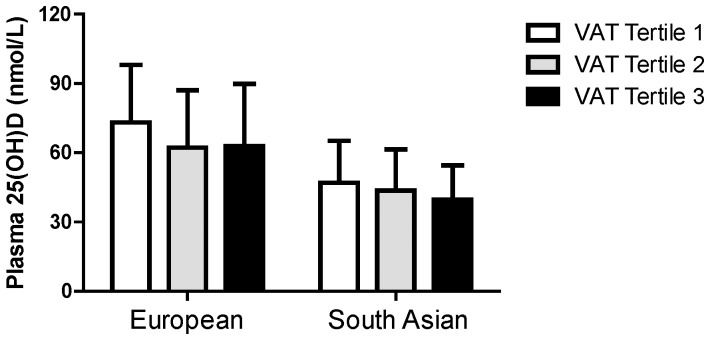
Plasma 25(OH)D concentrations by VAT tertiles in European and South Asian subjects. Plasma 25(OH)D concentrations were significantly different (*p* = 0.01) between VAT tertiles and significantly lower (*p*<0.001) in South Asians.

When percent total body fat, VAT, and SAT were included in the same linear regression model (adjusted for BMI, ethnicity, age, and season of blood collection) only VAT was significantly associated with plasma 25(OH)D concentrations in women (*p* = 0.005, standardized β = −0.289) and in men (*p* = 0.016, standardized β = −0.217) (Table 4). Ethnicity also remained a significant contributor (*p*<0.001) in these models. Plasma 25(OH)D concentrations stratified by VAT tertiles and ethnicity and shown in Figure 1, illustrating the lower plasma 25(OH)D concentrations in South Asian subjects compared to European subjects (*p*<0.001) and lower plasma 25(OH)D concentrations with increasing VAT tertiles (*p* = 0.01), as assessed by unadjusted general linear models.

**Table 4:** Relationship of plasma 25(OH)D with body fat compartments in men and women.

	Females		Men	
	β-coefficient (unstandardized)	Significance	β-coefficient (unstandardized)	Significance
BMI	0.149	*P* = 0.246	0.015	*p* = 0.881
Ln VAT	−0.289	*P* = 0.005	−0.217	*p* = 0.016
Ln SAT	−0.061	*P* = 0.672	0.020	*p* = 0.865
% Total body fat	−0.150	*P* = 0.237	−0.130	*p* = 0.258

Data were analyzed by linear regression models adjusted for ethnicity, age, BMI, and season of blood collection. VAT, SAT, and percent total body fat were included in the same model. Females and males were analyzed separately.

SAT, subcutaneous adipose tissue.

VAT, visceral adipose tissue.

## Discussion

The objective of this investigation was to assess the relationship of plasma 25(OH)D concentrations with anthropometric measures and body composition in a South Asian and a European population. A few smaller studies have reported low circulating 25(OH)D concentrations in South Asians [15]–[19]. We hypothesized that other factors, such as body composition, may also contribute to circulating 25(OH)D concentrations in South Asian populations. In our much larger population, we confirm that South Asians have lower plasma 25(OH)D concentrations and that plasma 25(OH)D concentrations are negatively associated with anthropometric measures and body fat distribution, with VAT displaying the strongest association with plasma 25(OH)D concentrations. Ethnicity remained a significant determinant of plasma 25(OH)D and we report for the first time that the lowest plasma 25(OH)D concentrations were found in South Asian subjects with the highest amount of VAT. Interestingly, plasma 25(OH)D concentrations remained significantly lower in the South Asian subjects even when VAT and total percent body fat differences were accounted for suggesting additional factors may contribute to the low circulating 25(OH)D concentrations in South Asian populations.

Our findings are in line with previous reports in American Caucasian subjects [10] and in Hispanic Americans and African Americans [13] that showed circulating 25(OH)D concentrations were independently associated with VAT and SAT. We confirm these findings in a large group of South Asians, a population with a unique phenotype of excess total body fat and VAT [20]. We also found that the negative association between low plasma 25(OH)D concentrations with percent body fat and SAT disappeared after taking VAT deposition into account. Despite the fact that VAT accounts for only 10% to 20% of total body fat [21], it appears to be the predominant region of adipose tissue associated with circulating 25(OH)D concentrations. The negative association of plasma 25(OH)D concentrations with VAT remained after controlling for other factors that influence plasma 25(OH)D concentrations such as age, date of blood collection, ethnicity, and BMI. Given that low plasma 25(OH)D concentrations are associated with CVD [22], this finding further highlights the importance of VAT as a contributor to CVD risk.

We previously reported that South Asians have a higher amount of VAT per kg body fat in comparison to the European subjects [20]. Despite this difference in VAT, plasma 25(OH)D concentrations remained lower in South Asian subjects compared to European subjects even after adjusting for VAT. This suggests that while VAT is associated with plasma 25(OH)D concentrations, it may not be the exclusive mechanism by which South Asian subjects have lower plasma 25(OH)D concentrations. South Asian skin colour may also be an important factor contributing to the low 25(OH)D concentrations observed in this population, although a recent study in South Asians reported no benefit of sunlight exposure on serum 25(OH)D concentrations [17].

South Asians have been reported to be at increased risk for CVD compared to other populations [23], [24], and this may in part be due to this combination of higher VAT levels and lower circulating 25(OH)D concentrations. A number of ways by which low circulating 25(OH)D concentrations are associated with CVD have been proposed and include: the direct role of vitamin D in the regulation of renin-angiotensin axis by suppressing the renin gene expression [25], the presence of vitamin D receptors on vascular smooth muscle and endothelial cells [26], and the direct influence of vitamin D status on hypertension [27].

The mechanism by which circulating 25(OH)D concentrations are associated with adipose tissue is not known. Vitamin D is a fat-soluble vitamin and as such may be sequestered by adipose tissue. A recent study reported that 25OHD concentrations in SAT increased following 12 wks of vitamin D3 or vitamin D2 supplementation [28]. Although the sample size in this study was small, these findings provide evidence in support of the concept that SAT accumulates 25OHD following vitamin D supplementation. However, what remains to be clarified is whether VAT also accumulates 25OHD and whether VAT is a determinant of plasma 25(OH)D concentrations or whether low plasma 25(OH)D concentrations promote VAT deposition. A recent study in women of European decent undergoing abdominal gynecological surgery also reported that serum 25(OH)D concentrations and dietary vitamin D intakes were negatively associated with VAT and negatively associated with adipocyte size in omental adipose tissue (VAT depot) [29]. A recent study reported that supplementation for 16 wks with calcium and vitamin D in over weight/obese individuals with adequate vitamin D status was associated with a reduction in VAT despite no significant differences in total body weight [30]. The separate effects of calcium and vitamin D could not be distinguished in this study, but these findings do provide additional evidence in support of a role for vitamin D in adipose tissue deposition. Furthermore, mice deficient in either the vitamin D receptor or 1α-hydroxylase, the enzyme responsible for the conversion of 25(OH)D to the biologically active 1,25-dihydroxyvitamin D3, were reported to have a lean phenotype when fed a high fat diet [31], further supporting a role for vitamin D in adipose tissue deposition.

An important strength of our study is that we have used sophisticated imaging techniques, such as DEXA and CT scanning, to quantify discreet regions of body fat composition. This enabled us to assess, and compare, the association of plasma 25(OH)D concentrations with several discrete indicators of body fat composition and distribution. Furthermore, we made our observations in two distinct ethnic groups, clearly illustrating the importance of ethnicity as a determinant of plasma 25(OH)D concentrations. A limitation of our study is that it is a cross-sectional observation. Further prospective studies are required to assess the relationship of plasma 25(OH)D concentrations with body fat composition and distribution over time and how this relates to the development of CVD in European and South Asian subjects. Another limitation of our study is that although we controlled for season of blood collection in our analyses, we do not have information regarding habitual sun exposure, which could contribute to the ethnic-specific differences in plasma 25OHD.

Overall we have shown that South Asian subjects have lower plasma 25(OH)D concentrations than European subjects and that plasma 25(OH)D concentrations are negatively associated with VAT levels, with the lowest plasma 25(OH)D concentrations observed in South Asian subjects in the highest tertile of VAT deposition. The association between VAT and plasma 25(OH)D concentrations was stronger than for SAT and percent total body fat, suggesting a distinct role for VAT with respect to plasma 25(OH)D concentrations and may in part, explain the lower plasma 25(OH)D concentrations in South Asians. This association may add yet another factor by which VAT increases CVD risk, however, this relationship needs to be explored in longitudinal studies. Further research delineating the molecular mechanisms by which VAT and vitamin D are metabolically related are required.

## Materials and Methods

### Study Participants

With approval from the Children's and Women's Health Centre of British Columbia, University of British Columbia and Simon Fraser University Research Ethics Boards, all participants provided written informed consent. This cross-sectional study is a subset of the M-CHAT cohort, which is a multi-ethnic population study that includes people of exclusive ancestry of Aboriginal, Chinese, European, and South Asian background residing in Vancouver, British Columbia, Canada [20]. Apparently healthy men and women (between 30 and 65 years of age) were purposefully recruited to ensure equal representation of obese (BMI ≥30.0 kg/m^2^), overweight (BMI 25.0 to 29.9 kg/m^2^) and desirable (BMI ≤25.0 kg/m^2^) BMI ranges for each sex-ethnic stratum. We excluded subjects who had undergone recent weight change (>2.2 kg in 3 mo), reported a previous diagnosis of CVD or significant comorbidity (such as HIV, immunocompromised condition, type 1 diabetes mellitus), were taking medications for CVD risk factors (ie, lipid-lowering, antihypertensive, or hypoglycemic medications), or had significant prosthetics or amputations. Subjects taking vitamin D supplements were not excluded. The current investigation is restricted to the 187 European (continental Europe, Ireland, and the United Kingdom) and 192 South Asian (Bangladesh, India, Nepal, Pakistan, and Sri Lanka) subjects from the M-CHAT cohort. Ethnicity and sex were self-reported.

### Anthropometric Assessment and Body Fat Distribution

Participant assessment has been previously described [20], [32]. Weight was assessed in subjects with light street clothing, footwear removed, and pockets emptied. No corrections for clothing were made. Weight and height measures were taken to determine BMI [mass(kg)/height(m)^2^] and waist circumference (WC) was assessed by taking an average of two measurements against the skin at the point of maximal narrowing of the waist. Abdominal adipose tissue was quantified by computed tomography (CTi Advantage, General Electric, Milwaukee, WS, USA) as previously described [32], [33]. A 10-mm cross-sectional slice taken from the L4/L5 intervertebral disc with an attenuation range of −190 to −30 Hounsfield units was used to identify adipose tissue. To compute surface areas from the CT-scans, SliceOmatic 4.2 medical imaging software was used (SliceOmatic v4.2. Tomovision, Montreal, Canada). Total abdominal adipose tissue was defined as all the observed adipose tissue in the CT scan slice and VAT as the adipose tissue found within the inside edge of the abdominal muscle wall. Subcutaneous adipose tissue (SAT) was calculated as the difference between total adipose tissue and VAT. Whole body total adipose tissue was quantified by dual-energy X-ray absorptiometry (DEXA) with a Norland XR-36 scanner (Norland Medical Systems, White Plains, NY, USA). Total percent body fat was calculated by dividing the whole body total adipose tissue (as quantified by DEXA) by total body weight.

### Vitamin D Status

Fasting plasma from EDTA-treated blood was used for assessing plasma 25(OH)D. Plasma 25(OH)D was quantified by BC Biomedical Laboratories Ltd (Surrey, BC, Canada) using a *DiaSorin* LIAISON® 25-OH Vitamin D TOTAL Assay, a competitive chemiluminescence immunoassay used for the quantitative determination of both 25(OH)D2 and 25(OH)D3 metabolites [34]. BC Biomedical Laboratories Ltd.

### Statistical Analyses

Normally distributed continuous variables are reported as means ± standard deviations, and categorical and binary factors as percentages. Based on variable distribution using histograms, VAT and SAT were not normally distributed and were transformed using the natural log prior to statistical analyses. These variables are presented in their untransformed values as medians and the 25th and 75th percentile values. Univariate comparisons between South Asians and Europeans were made by ANOVA for continuous variables and Pearson's Chi-squared tests for categorical variables. We did not adjust for multiple comparisons. Linear regression was used to assess univariate and multivariate associations between plasma 25(OH)D and each of the variables of interest (BMI, waist circumference, waist-to-hip ratio, total abdominal adipose tissue, VAT, SAT and percent body fat). Subsequent models were adjusted for BMI, age, ethnicity, sex, and season of blood collection. We first tested for interactions between sex and season of blood collection, sex and ethnicity, and ethnicity and season of blood collection and found a significant interaction (p<0.05) between sex and season of blood collection only. As such, data for males and females were analyzed separately. Linear regression was also used to identify the strength of the relationship between the various body composition measures (percent total body fat, VAT, and SAT) with 25(OH)D concentrations adjusted for BMI, ethnicity, age, and season of blood collection. Analyses were conducted using SPSS software version 18.0 (Chicago, IL, USA).
